# An Unexpected Turn: Management of a Life-Threatening Infection Following Liposuction Procedure

**DOI:** 10.7759/cureus.53795

**Published:** 2024-02-07

**Authors:** Marwa Morgom, Doaa M Eisa, Hanna Ali, Leena Saeed

**Affiliations:** 1 Emergency Medicine, Tripoli Central Hospital, Tripoli, LBY; 2 Internal Medicine, Hamad Medical Corporation (HMC), Doha, QAT; 3 General Practice, Hamad General Hospital, Doha, QAT

**Keywords:** recurrent necrotizing fascitis, heart failure, abdominoplasty, liposuction complication, necrotizing fascititis

## Abstract

This case report presents a rare case of necrotizing fasciitis (NF) following liposuction and lipofilling surgery in a young woman. Despite prompt diagnosis and aggressive management with multiple debridements, broad-spectrum antibiotics, and supportive care, the patient experienced a protracted course with severe complications, including intra-abdominal collection recurrence, heart failure, and sepsis. The presence of resistant bacteria (extended-spectrum beta-lactamases (ESBLs)-producing *Escherichia coli *and methicillin-resistant *Staphylococcus aureus* (MRSA)) further challenged the treatment. This case highlights the importance of early recognition and aggressive management of NF, particularly in patients with risk factors following cosmetic surgery. In addition, it raises awareness of the potential for heart failure as a complication in this context and warrants further investigation.

## Introduction

Necrotizing fasciitis (NF) is a soft tissue infection that progressively spreads through the skin, subcutaneous tissue, and fascia. It is commonly caused by bacterial organisms, such as *Streptococcus pyogenes*. The causative agent reaches the bloodstream by invading through wounded or intact skin, and post-surgical status is a strong risk factor for developing NF. It is mainly diagnosed clinically by the presence of a variety of findings, such as insidious skin lesions, severe unproportioned pain, and multi-organ damage. Management options include surgical excision of the affected tissues, supportive care, and combined antibiotic therapy. Nevertheless, mortality rates remain high especially after surgical interventions as it may reach up to 30-50% [1,2].

Liposuction and lipofilling are types of cosmetic surgical procedures characterized by removing adipose tissue from certain areas and transferring it to where the patients desire to obtain greater volume to improve body contour. This is obtained by using suction cannulas through small incisions in the skin with minimal scarring and recurrence. The complication rate for this procedure is estimated at 5%, and the occurrence of infections, such as NF, is considered rare. Its incidence is reported as 0.7% of cases when multiple procedures are performed along with liposuction [2,3]. In our case, we report a 35-year-old lady who developed NF following a liposuction procedure. She experienced multiple complications, including intra-abdominal collection and heart failure. We will emphasize the importance of early recognition of NF and its immediate treatment.

## Case presentation

**Figure 1:**
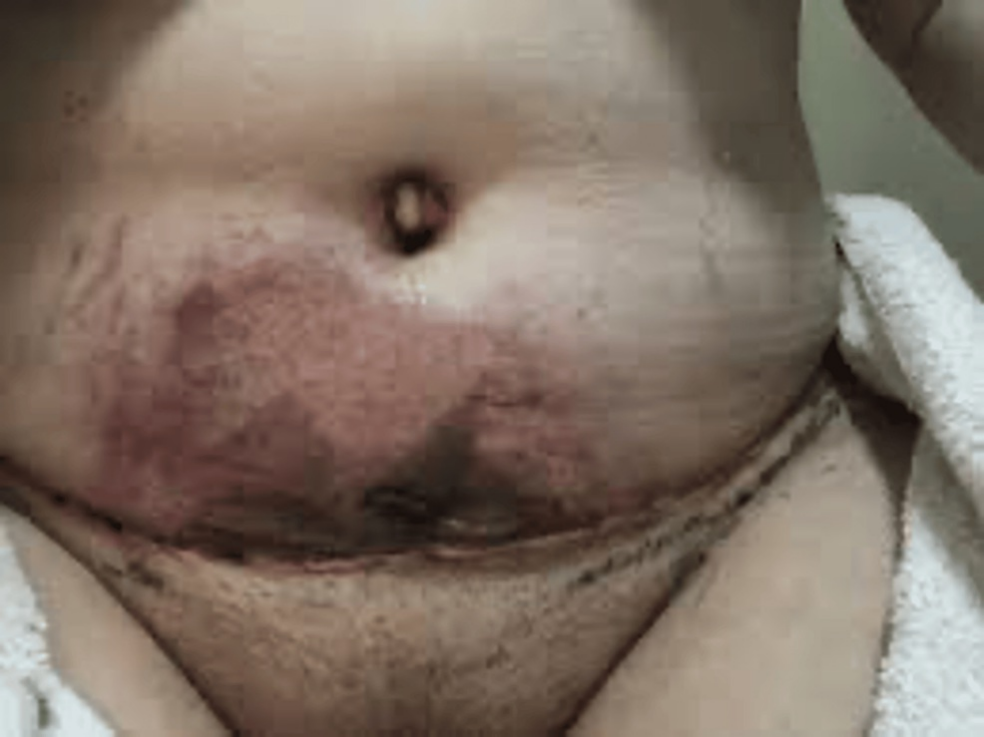
Skin changes post surgery at the first emergency department (ED) visit

This case involves a 35-year-old lady who presented to the emergency department (ED) with a complaint of fever and fatigue after undergoing a body contouring surgery that was done in Turkey 10 days prior to the presentation. Her surgery involved liposuction for the abdomen, flanks, and arms, and lipofilling for trochanters and buttocks. She had an episode of fever of 38°C after she arrived from Turkey, and she immediately reported to the ED. She also complained of excessive thirst and pain over the surgical sites, especially in the abdomen, back, bilateral thighs, and upper arms. In the ED, she was found to be sleepy with a hyperdynamic pulse. Therefore, she was shifted to the resuscitation area. Upon clinical examination, she was wearing a pressure garment, and a dark discoloration with blistering was noted over the central lower abdomen (Figure 1). It was associated with erythema and warmth. Minimal induration and bruising were noted on bilateral flanks. The right upper arm dressing was producing a green discharge. The rest of the dressings were intact and clean without discharges, except for scattered bruising over the back wound.

**Figure 2:**
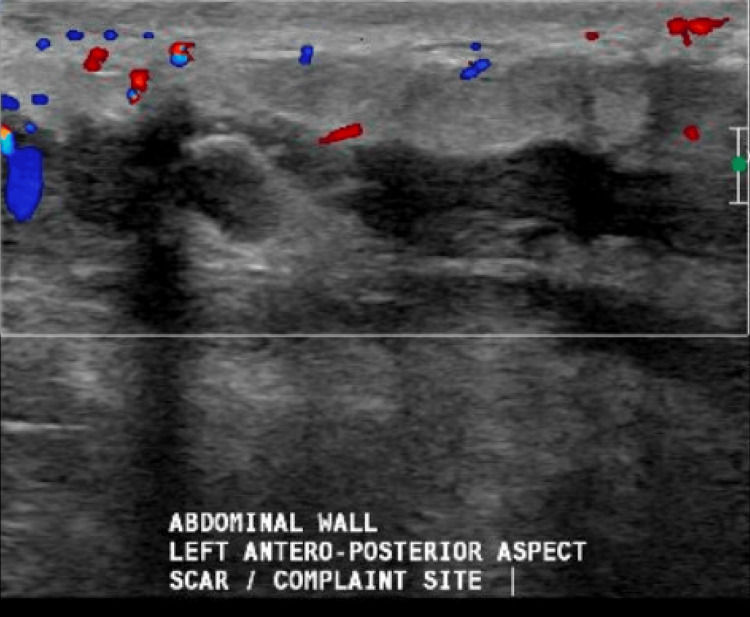
Ultrasound of the abdominal wall during the first visit to ED reflecting pus accumulation

**Figure 3:**
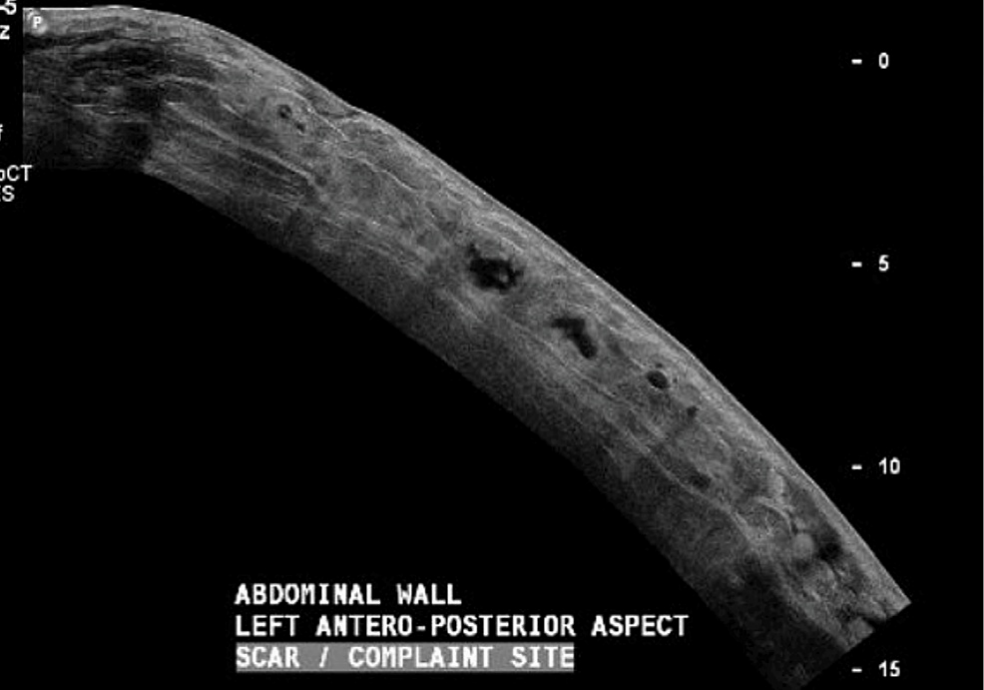
Another view of the ultrasound reflecting subcutaneous tissue involvement with gas accumulation

**Figure 4:**
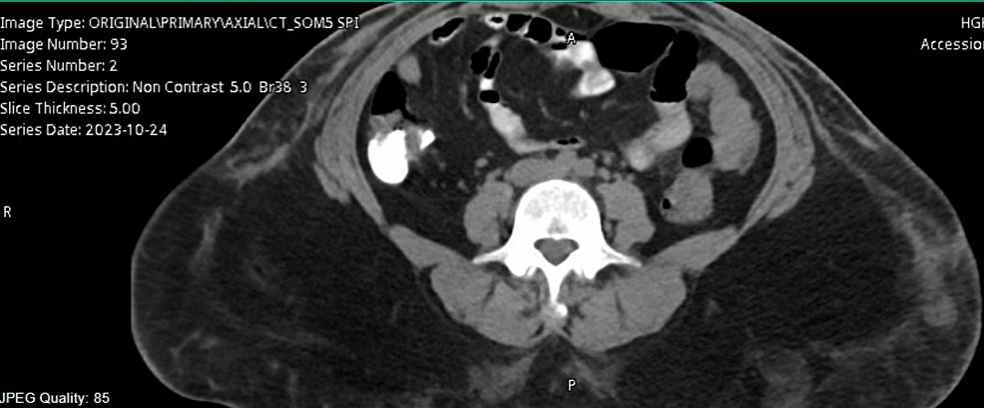
CT at the first visit to ED showing subcutaneous tissue swelling, debris accumulation

Her vital signs were stable apart from fever and tachycardia (Table 1), and her serum lactate was 2.4 mmol/L. Hence, fluid resuscitation and meropenem 1 gm q8H were started. She was also catheterized for urine output monitoring (Table 2). Plastic surgery on-call was contacted for the possibility of necrotizing fasciitis, and an emergency ultrasound abdomen multiple views performed, revealing an abdominal wall collection and gas accumulation, followed by CT abdomen with contrast (Figures 2, 3, 4)*.* The plastic surgeon advised admission to the Surgical Intensive Care Unit (SICU) after operating room (OR) assessment.

**Table 1:** Vital signs for the patient at the time of presentation, during stay, and until discharge ED: emergency department, SICU: Surgical Intensive Care Unit, OT: occupational therapy

	At the first ED visit	Post OP/debridement/first day at SICU	SICU 8th day (post second OT intervention)	At SICU day 10 post 3rd OT intervention	At SICU day 13 post 4th OT intervention	At cardiology consultation day	At discharge day
Temperature	38 °C	37.2 °C	36 °C	36.5 °C	37 °C	36.7 °C	36.5 °C
Blood pressure	100/60 mmHg	120/70 mmHg	130/80 mmHg	140/90 mmHg	140/80 mmHg	140/90 mmHg	120/69 mmHg
Respiratory rate	27 cycle/min	19 cycle /min	18 cycle /min	17 cycle /min	18 cycle /min	25 cycle /min	18 cycle /min
Oxygen saturation	96% on room air	95% on nasal cannula 1 liter	97% on room air	97% on room air	96 % on nasal cannula 1 liter	95% on nasal cannula 2 liter	97% on room air
Heart rate	107 beats/min	90 beats/min	91 beats/min	80 beats/min	95 beats/min	110 beats/min	70 beats/min

**Table 2:** IV fluid replacement protocol at the ICU

Days	Input	Output
Day 1 post-intervention (first surgery )	IV fluid dextrose 5%/0.45NS 125 ml/hr maintenance	Urine output 60 ml/hr, balance 2800 ml 24/hr
Post 2nd intervention	IV fluid dextrose 5%, 0.45 NS, 125 ml/hr maintenance	Urine output 80 ml/hr, balance 3000 ml 24/hr
Post 3rd intervention	IV fluid dextrose 5%, 0.45 NS, 125 ml/hr/hr maintenance	Urine output 80 ml/hr, balance 3000 ml 24 /hr
Post 4th intervention	IV fluid dextrose 5%, 0.45 NS, 125 ml/hr/hr maintenance	Urine output 70 ml/hr, balance 3000 ml 24 /hr
At the day of cardiology consultation	IV fluid dextrose 5%, 0.45 NS, 100 ml/hr/hr maintenance	Urine output 90 ml/hr, balance 3000 ml 24 /hr

The patient underwent abdominal wall debridement and vacuum-assisted closure (VAC) application under general anesthesia, and after six days, a second-look debridement with VAC application was performed. A third debridement and wash of the raw area in the anterior abdominal wall was done with a changing of VAC dressing, split-thickness skin graft (STSG) application, and primary closure of the wound at the left flank one week later from the second intervention. Tigecycline 100 mg intravenous (IV) infusion initially started, and then the dose changed to 50 mg IV infusion q12hr for five to 14 days. In addition, clindamycin 20-40 mg/kg/day IV was divided into q6-8hr as her tissue cultures showed extended-spectrum beta-lactamases (ESBLs)-producing *E. coli* and methicillin-resistant *Staphylococcus aureus* (MRSA), and she remained stable with daily dressing. Fluid maintenance at the ICU stay period is mentioned in Table 2.

**Figure 5:**
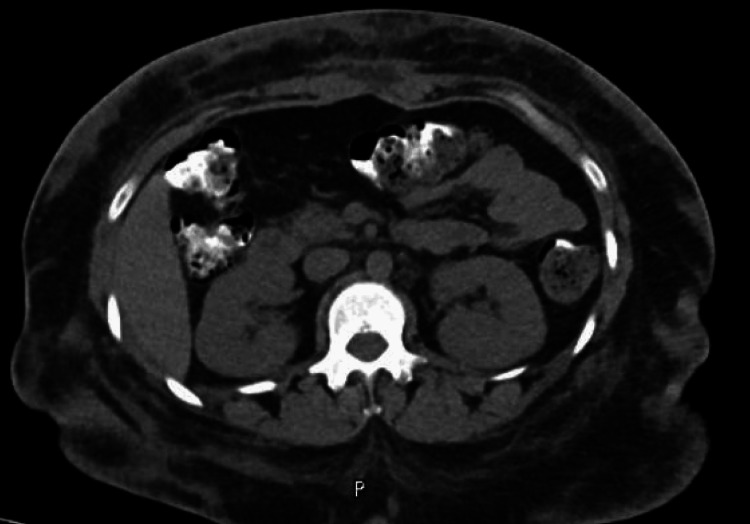
CT showed re-accumulation of access and necrotic tissue with classification and post-operative changes

**Figure 6:**
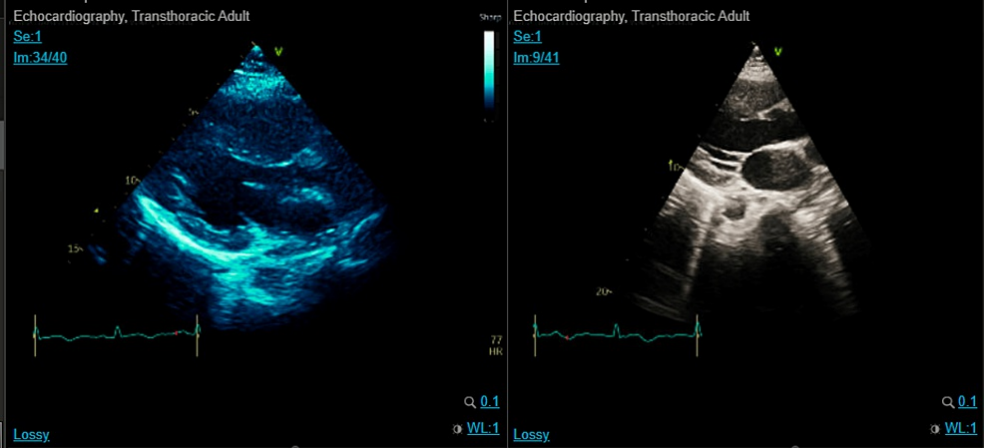
Echo showed an acute heart failure image with global hypokinesia

On day 25 of admission, she underwent her last operative intervention due to residual necrotizing. Tissue and pus accumulation was performed (Figure 5). After it, at the ICU, she developed shortness of breath, cough, tachycardia, and raised the jugular venous pressure (JVP). Basal fine crackles and gallop rhythm were noted upon auscultation. CT pulmonary angiogram was negative for pulmonary embolism. Her labs showed elevated troponin and pro-B-type natriuretic peptide (BNP) (Table 3), and echo revealed reduced systolic LV function with a biplane left ventricular ejection fraction (LVEF) of 30%, which was normal on the initial baseline echo (Figure 6).

**Table 3:** Labs showing patient status at the ICU

Group	Detail	Value w/ units	Flags	Normal range
General hematology	Hgb	8 gm/dL	Low	12.0-15.0
Blood chemistry	NT pro-BNP	8,035 pg/mL	N/A	
Blood chemistry	Troponin-T HS	80 ng/L	High	3-10
Blood chemistry	Creatinine	34 umol/L	Low	44-80

The cardiologist's diagnosis was a newly detected heart failure with reduced ejection fraction (HFrEF) with no clear reason, but it is likely to be a metabolic, septic, or viral infection in origin and less likely attributable to coronary artery disease (CAD). Thus, she was started on furosemide 40 mg BID, valsartan 40 mg daily, spironolactone 12.5 mg daily, aspirin 100 mg daily, and bisoprolol 2.5 mg daily with the advice of repeating echo in the cardiology outpatient clinic four to six weeks after discharge. The patient significantly improved after she started her medications, and she was discharged after two days after her transfer to the surgical ward. She was discharged on non-steroidal anti-inflammatory drug (NSAID) analgesia with cardiology medications and outpatient follow-up with plastic and cardiology clinics.

Seven days post-discharge, she presented to the ED with two days of history of severe pain in the upper abdomen with multiple spikes of high-grade fever reaching up to 40°C. It was associated with chills, nausea, loss of appetite, generalized fatigue, and weight loss of 20 kg in 15 days. Upon assessment in the ED, she was hemodynamically stable, except for a fever of 38°C. Repeated labs were all normal, and repeated CT abdomen showed "anterior and posterior abdominal wall collections" (Figure 6). Meropenem and clindamycin were restarted on same previous doses along with anidulafungin 200 mg loading dose and then tapered to 100 mg. The Plastic Surgery and Infectious Diseases teams were consulted, and as a result, ultrasound-guided aspiration of the abdominal wall collection was decided. During the intervention, 10 cc of pus was collected. The patient received antibiotics and antifungals in the surgical ward for seven days. After her fever subsided, she was safely discharged with analgesia and scheduled for follow-up in a high-risk plastic outpatient clinic within five days.

## Discussion

NF is a fatal infection that affects soft tissues and fascia underneath the affected region. The most involved areas are the lower extremities, abdomen, and perineum. The NF annual incidence rate has been reported in a few cohorts and case reports with a range from 0.72 to 9.2 per 100,000 person-years. In addition, the mortality rate of NF is quite high, as mentioned in some studies, with an annual death rate of 32.2% reported. In the USA, there are 4.8 deaths per 1,000,000 cases [4,5,6,7,8]. 

The diagnosis of NF can be challenging as it is mainly clinical, and a high level of suspicion needs to be there to detect it early. Patients with multiple comorbidities, such as diabetes mellitus, liver cirrhosis, advanced age, and immunocompromised patients, are at a higher risk. The Laboratory Risk Indicator for Necrotizing Fasciitis (LRINEC) scoring system was created to aid in the assessment and early recognition of the disease as it helps to differentiate NF from other soft tissue infections. Radiological diagnostic methods can also aid in diagnosis; however, it should not delay the definitive management as it is associated with high mortality. The diagnosis of NF should be considered if the patient looks sick or the pain is disproportionate to the clinical picture. In addition, skin changes and crepitation may not be clear at the early phase of the disease. However, in the later stages of the disease, the skin may become numb due to nerve involvement in disease progression [8,9,10,11].

Liposuction and heart failure

Abdominal liposuction is considered a routine procedure that can be performed in outpatient settings. However, deadly complications may occur. Those may include bowel and visceral perforation during the procedure, peritonitis, pulmonary embolism, NF, and shock. Nevertheless, there is no clear reason for the connection between heart failure and liposuction procedures [12]. In our case report, we present this complication and highlight it as a possible complication that should be considered while dealing with such cases.

Management of NF 

First, proper successful debridement with dressing and negative pressure wound therapy should be done. This therapy is an important adjuvant method in the treatment of wounds and accelerates wound healing and repair until tissue construction can be used. Second, the treatment of NF necessitates IV administration of broad-spectrum empirical antibiotics according to the microbiological classification.

There are three types of NF: Type I, which has a greater prevalence and corresponds to polymicrobial NF (presence of two or more infective agents, which can be gram-positive, anaerobic, or gram-negative), mainly affects patients with comorbidities. Type II occurs less frequently and mainly affects young patients or those without comorbidities. It is caused by the beta-hemolytic *Streptococcus* group A, *S. aureus*, or an association between both. Type III is extremely rare, and it is the most aggressive type. It is associated with sepsis that may result in multi-organ failure in less than 24 hours. It is commonly caused by *Vibrio vulnificus*, which can be found in seawater and marine animals.

Vancomycin combined with carbapenem is the best antibiotic of choice [13]. In the present case, “meropenem and clindamycin” were the empirical choices of medications, and they were continued after culture and sensitivity results.

## Conclusions

NF is a fatal infection that does not occur commonly and is rarely reported after cosmetic surgeries. However, if it happens, high mortality is expected. In our case, NF is caused by resistant organisms following liposuction and lipofilling reconstructive surgeries. Multiple morbidities occurred, such as sepsis, recurrence of the abdominal collection, and heart failure. Early detection of the condition and prompt starting of the antibiotics are crucial for the patient management.
